# Intensive social cognitive treatment (can do treatment) with participation of support partners in persons with relapsing remitting multiple sclerosis: observation of improved self-efficacy, quality of life, anxiety and depression 1 year later

**DOI:** 10.1186/s13104-016-2173-5

**Published:** 2016-07-29

**Authors:** Peter Joseph Jongen, Marco Heerings, Rob Ruimschotel, Astrid Hussaarts, Lotte Duyverman, Anneke van der Zande, Joyce Valkenburg-Vissers, Maarten van Droffelaar, Wim Lemmens, Rogier Donders, Leo H. Visser

**Affiliations:** 1 Department of Community and Occupational Medicine, University Medical Centre Groningen, Antonius Deusinglaan 1, 9713 AV Groningen, The Netherlands; 2 MS4 Research Institute, Ubbergseweg 34, 6522 KJ Nijmegen, The Netherlands; 3 National Multiple Sclerosis Foundation The Netherlands, Mathenesserlaan 378, 3023 HB Rotterdam, The Netherlands; 4 Medical Psychiatric Centre PsyToBe, Metroweg 50, 3083 BB Rotterdam, The Netherlands; 5 Fysiotherapie Maaspoort, Maaspoortweg 182, 5235 KB ‘s-Hertogenbosch, The Netherlands; 6 Department for Health Evidence, Radboud university medical centre, P.O. Box 9101, 6500 HB Nijmegen, The Netherlands; 7 Multiple Sclerosis Center Midden Brabant, EZT, Location St. Elisabeth, Hilvarenbeekseweg 60, 5022 GC Tilburg, The Netherlands; 8 University of Humanistic Studies, Kromme Nieuwegracht 29, 3512 HD Utrecht, The Netherlands

**Keywords:** Self-efficacy, Multiple sclerosis, Autonomy, Participation, Quality of life, Depression, Anxiety, Fatigue, Social, Cognitive

## Abstract

**Background:**

In persons with multiple sclerosis (MS) self-efficacy positively affects health-related quality of life (HRQoL) and physical activity. In a previous study we observed that 6 months after an intensive 3-day social cognitive treatment (Can Do treatment) with the participation of support partners, self-efficacy and HRQoL had improved in persons with relapsing remitting MS (RRMS). Given the chronic nature of the disease, it is important to know whether these beneficial changes may last.

**Methods:**

Can Do treatment was given to 60 persons with MS and their support partners. At baseline and 12 months after treatment self-efficacy control, self-efficacy function, physical and mental HRQoL, anxiety, depression and fatigue were assessed via self-report questionnaires. Differences were tested via a paired *t* test.

**Results:**

Of the 57 persons with MS that completed the baseline assessment and the 3-day treatment, 38 filled in the 12th month questionnaires (response rate 66.7 %), 22 with RRMS and 14 with progressive MS. In the RR group self-efficacy control had increased by 20.2 % and physical HRQoL by 15.0 %, and depression and anxiety had decreased by 29.8 and 25.9 %, respectively (all P < 0.05); the changes in mental HRQoL (+17 %) and fatigue (−20 %) failed to be statistically significant (P = 0.087, P = 0.080, respectively). In the progressive group no changes suggestive of improvement were seen.

**Conclusions:**

The findings suggest that a 3-day intensive social cognitive treatment (Can Do treatment) with the participation of support partners may have long lasting beneficial effects on the self-efficacy and HRQoL in persons with RRMS; and that improvements in anxiety and depression, not seen in the 6-month study, may yet develop at 12 months.

## Background

Multiple sclerosis (MS) is a chronic disease of the central nervous system pathologically characterized by immune-mediated inflammation, demyelination and axonal degeneration. In most persons with MS the first symptoms occur between 20 and 45 years of age, and the initial course is characterized by relapses, followed by complete or incomplete recovery: relapsing-remitting MS (RRMS). The symptoms and disabilities are highly variable and unpredictable. Despite disease modifying treatment most people with RRMS convert to the secondary progressive phase (SPMS), for which no treatment is available. The response to disease modifying treatment in RRMS varies between persons and is difficult to prognosticate.

Self-efficacy is a psychological concept that refers to the degree in which a person is confident to complete tasks and reach goals in specific situations [1–3]. The belief in one’s ability to produce effects or outcomes one wants is a core component in social cognitive theory, in which psychosocial functioning is determined by reciprocal interactions between personal factors, behaviour, and the environment [1–3]. Self-efficacy is influenced by experience, social persuasion, and physiological factors [1–3]. Self-efficacy itself may affect human function in various ways. First, by influencing choices regarding behaviour: people generally avoiding tasks where self-efficacy is low, but undertaking tasks where self-efficacy is high [4, 5]. Second, by affecting motivation: people with high self-efficacy are more likely to make efforts to complete a task, and to persist longer in those efforts, whereas those with low self-efficacy will tend toward discouragement and giving up [4–6]. Third, self-efficacy has effects on thought patterns and responses: low self-efficacy can lead people to believe tasks to be harder than they actually are, which often results in poor planning and increased stress [4, 5].

In chronic conditions the relationship between self-efficacy and psychological well-being has been convincingly documented [3, 7]. Self-efficacy is also one of the most consistent determinants of physical activity across populations, including those with MS [8]. Most people with (PwMS) become increasingly disabled in the course of the disease, with disabilities negatively affecting their independence, and the negative experience of losing independence negatively affecting self-efficacy. In fact, MS is associated with a large reduction in physical activity behaviours, and evidence indicates that this reduction correlates with self-efficacy [9]. It has also been demonstrated that in PwMS self-efficacy not only has a positive relationship with physical activity [10–12], but also with physical and psychological health-related quality of life (HRQoL) [13–15], and psychological adjustment [7]; and that it is negatively associated with depression [6].

Randomized controlled trials have shown that various treatment modalities may improve self-efficacy in patients with chronic conditions. In persons with persistent neck pain a multi-component self-management group intervention resulted in a 2-year improvement in pain-related self-efficacy [16], and in a 1-year study in patients with chronic low back pain both mindfulness-based stress reduction and cognitive-behavioural therapy had a positive effect on self-efficacy [17]. In rheumatoid arthritis patients a 32-week study showed that needs-based patient education did improve arthritis-related self-efficacy [18], and in older adults with osteoporosis balance training with multi-task exercises improved fall-related self-efficacy in a 12-week study [19]. As far as MS is concerned, Rigby et al. [20] showed in a 12-month randomized controlled trial that group cognitive-behavioural sessions supported by an information booklet, and non-structured social discussion sessions supported by an information booklet, both resulted in improved MS-related self-efficacy.

In 2014 we reported the results of an observational study in PwMS on the effects of an intense, multidisciplinary, 3-day, social cognitive Can Do treatment program with the participation of support partners [21]. The concept of the Can Do treatment is based on the biopsychosocial model of somatisation, developed by Rosen, Kleinman and Katon [22], augmented by elements from clinical psychotherapy. According to the biopsychosocial model, illness behaviours like somatic symptom presentation and medical help seeking occur in reaction to underlying life stressors, e.g. unemployment or marital conflict [22]. Physicians and the medical care system often play a significant role in reinforcing somatisation by patients [22]. The identification of individual stressors and their elimination or mitigation are believed to result in decreased somatisation and help seeking, and in increased self-efficacy and autonomy. The supposedly therapeutic elements in the Can Do approach are group interactions, the accent on potentials instead on limitations, the participation of the support partner, the necessity to take initiatives and actions, obtaining information leading to adjustment of the self-image, the presence of a multi-disciplinary team guaranteeing that all aspects of living with a chronic diseases are considered, and every action depending on an explicit choice. Crucial to the Can Do treatment is that patients and their support partners are continuously encouraged to make choices and to take action, and are thus stimulated to make optimal use of their self-efficacy.

We found that 6 months after Can Do treatment, relapsing remitting (RR) patients showed a 24.8 % increase in self-efficacy control (primary outcome) and 22.3 and 17.6 % increases in mental and physical HRQoL (secondary outcomes), respectively [21]. In contrast, progressive patients only showed an increase in mental HRQoL by +11.5 % at 1 month. However, given the chronic, and as yet incurable, nature of the MS disease process, it is important to be informed on eventual improvements occurring after Can Do treatment in the longer term. Therefore, to explore whether the beneficial changes seen at 6 months could also be observed at 1 year we performed the present study. Here we report the results.

## Methods

### Can Do treatment

#### Concept

The Can Do treatment aims to uncover and promote existing capabilities, with the notion ‘stressor’ as central concept. The treatment is primarily a sociologically oriented approach. It tries to identify stressors that confine PwMS to their physical, psychological or social roles. To reduce these stressors, the treatment is based on five principles: identification and reduction of existing stressors; client-centeredness; inclusion of the partner or another significant informal caregiver; group sessions; and self-reliance, autonomy, and acceptance as central themes. Accordingly, the Can Do treatment focuses on the exploration of stressors that confine PwMS to their disease and their limitations; reduces relevant stressors; makes participants to explore and exceed their own boundaries; and creates new personal boundaries by optimally using the existing potential. To give the capabilities of each individual a realistic framework the central mottos are ‘Can’, ‘Will’, ‘Choose’, ‘Open up to others’, and ‘Do’. The treatment’s message is that by exploring their boundaries PwMS become more aware of their faculties and improve their self-management, which leads to a higher awareness of potentials and a better communication with health care professionals.

#### Components

The treatment includes (1) large group sessions, (2) small group sessions, (3) consultations, (4) a theatre evening, and (5) start of the day with a joint activity (optionally).

*Large group sessions* Plenary sessions in which the participants make optimal use of their potentials, learn how to support and encourage each other, and experiment on how to give the required feedback to the multidisciplinary team. Group sessions including half the participants in which the participants examine and identify the stressors that have to be addressed most, and formulate realizable individual aims (one or two).

*Small group sessions* The small group sessions form the actual treatment. Depending on the individual goals the participants sign up for the training groups ‘body’, ‘feeling’ or ‘life’, to work out their aims and to experiment whether they can reduce their stressors. The body sessions explore the physical capabilities and are coached by a physiotherapist. The feeling sessions explore the emotional potential and are coached by a psychiatrist and a psychiatric nurse. The life sessions explore the capabilities relating to the daily living with MS and are coached by a neurologist, a registered nurse specialized in MS, and a person with MS. Moreover, there are relaxation sessions: the Yoga session focusing on body experience and relaxation, and the Physical session focusing on relaxation through physical strain. Participants choose between the various small group sessions autonomously.

*Consultations* After having identified and formulated (large group sessions) the individual stressors and aims, the participants sign in for one or more group consultations, to verify whether their aims are realistic, by asking the team members for aim-related medical information.

*Theater evening* On the informal theater evening the participants practice to change roles and to show their potentials by openly experimenting. They do their best to perform before each other and the team. The collective performance increases the group cohesion and learns the participants to find an equilibrium between consuming and action.

*Common activity at the start of the day* During an optional common activity (walk in the woods) at the start of the day the participants experiment with physical challenges and with their energy management.

#### Multidisciplinary team

The multidisciplinary consisted of a psychiatrist, psychiatric nurse, neurologist, MS Nurse, Physiotherapist, Yoga Teacher, and a person with MS. The team members stimulate, defy and confront the participants to explore and extend their boundaries. Apart from the consultations, the team keeps to coaching, motivating and activating the participants. By participating in all large group sessions the team members familiarize themselves with the participants’ stressors and goals. During the consultations they have a professional and informative role and in the small group sessions they focus on their own areas of interest. During a tip time at the end of the day the team members evaluate the sessions, inform each other on the participants’ progresses and obstacles, discuss whether the participants make optimal use of their capabilities, and monitor to what degree the individual goals are being attained.

### Study design and organization

The study was exploratory and uncontrolled. The Can Do treatment was given during 3 days. Each treatment course was given to 10 PwMS and their significant support partner (partner or informal caregiver). The inclusion criteria were: diagnosis definite MS, relapse free in the last 4 weeks, and able and willing to participate in the Can Do treatment and the study-related assessments. Data were obtained by the use of paper-and-pencil self-report questionnaires, sent by regular mail 1–4 weeks before and 12 months after the treatment, accompanied by a stamped return envelope addressed to the MS4 Research Institute (groups 1 and 2; baseline assessment in groups 3–6) or by online versions of the questionnaires (month 12 assessment in groups 3–6). As to the latter, after having given informed consent the patients received a personal code and logged on to the website of the MS4 Research Institute, to choose a username and password. The online assessments were performed using the LimeSurvey software, an open source online application. There was no testing of the MS4 Research Institute’s platform for this study since it was already being used in various research projects. The items of the questionnaire were fixed. The responses were automatically captured. To protect the personal data from unauthorized access various mechanisms were used to comply with European Union regulations concerning online medical data, including the use of a personal username and a strong password, separation in the database of personal information from the answers to the questions, each screen having a username and password protection, VPN tunnelling, 256-bits encryption, and the encryption of the participants’ identities via unique 10 digits codes. Automated completeness checks were done before questionnaires could be submitted. The respondents saw an overview of all questions and answers before submission and they could change the answers before submitting. After submission changes were no longer possible. Only completed questionnaires were analyzed. The help desk (MH) contacted respondents by phone when they had not completed the questionnaires within 1 week after the scheduled date, in order to remind them.

### Outcome measures

#### Primary outcomes measures

Self-efficacy was assessed by the multiple sclerosis self-efficacy scale (MSSES) [23], which consists of two 9-item subscales of function and control. Each item is scored on a Likert-like scale form 10 (very uncertain) to 100 (very certain) and addition of the respective item scores yields the MSSES function and control scores, both ranging from 90 (minimum) to 900 (maximum). The MSSES function subscale measures confidence with functional abilities, whereas the MSSES control subscale measures confidence with managing symptoms and coping with the demands of illness [23].

The impact on participation and autonomy (IPA) questionnaire is a 32-item self-report instrument for the quantification of limitations in participation and autonomy in persons with chronic health conditions [24, 25]. The IPA Limitations subscale assesses perceived limitations in participation and autonomy in relation to 32 different life situations across five subscales [24–26]. Items are rated on a 5-point scale from 0 (very good) to 4 (very poor), and a higher score indicates a higher limitation to participation and autonomy. The IPA Problems subscale examines the extent to which these limitations are experienced as problematic, by assessing nine different areas of participation and autonomy [24–26]. The perceived problems are graded on 3-point scale ranging from 0 (no problem) to 2 (severe problems), and a higher IPA Problems score indicates a greater experience of problems [24–26].

#### Secondary outcome measures

HRQoL was assessed by the Multiple Sclerosis Quality of Life 54-Items (MSQoL-54) questionnaire [27]. A physical and a mental dimension underlie the MSQoL-54: the Physical and Mental domains [27]. Scores for each domain range from 0 to 100, where higher values indicate better HRQoL. Anxiety and depression were measured by the Hospital anxiety and depression scale (HADS) [28]. The HADS consists of two subscales, one for anxiety and one for depression, each comprising seven questions; each question scores 0–3 points and addition of the seven question scores yields the anxiety and depression subscale scores [28].

Fatigue was measured by the multiple sclerosis fatigue impact scale 5-Items Version (MFIS-5) [29]. Answers to each question are rated on a 5-point scale from 0 to 4; the MFIS-5 total score consists of the sum of the raw scores on these 5 items and ranges from 0 to 20, where higher scores indicate more experienced fatigue [29].

### Ethical aspects

The study protocol was presented to the ethical review board “Medisch-Ethische Toetsing Onderzoek Patiënten” (METOPP), Tilburg, The Netherlands. Centrale commissie mensgebonden onderzoek (CCMO) (Central Committee on Research Involving Human Subjects) number: NL36051.028.11 (http://www.ccmo.nl/en). The committee concluded that a review was not indicated, as the study did not qualify for being tested according to the Dutch Medical Research Involving Human Subjects Act of 1999 (http://www.wetten.overheid.nl/BWBR0009408) [30]. PwMS interested in participation were informed on the study and its procedures, had their questions answered, and underwent a screening interview and inclusion procedure. No incentives were offered. PwMS were included after they had given their written or online consent. The study was performed in agreement with the Declaration of Helsinki (Ethical Principles for Medical Research Involving Human Subjects version 2013; 64th World Medical Association General Assembly, Fortaleza, Brazil, October 2013) (www.wma.net) and the Wet medisch-wetenschappelijk onderzoek met mensen (WMO) (Dutch Medical Research Involving Human Subjects Act) (www.wetten.overheid.nl/BWBR0009408).

### Data analysis

For all outcomes the absolute values at baseline and at 12 months after the Can Do treatment are described as mean with standard deviation (SD), and the percentage changes from baseline at 12 months are described as mean with standard error of the mean (SEM). Given the explorative nature of the study, we compared each outcome with its baseline value by using a paired Student t-test. To evaluate the degree and clinical relevance of eventual changes we tested for each outcome the percentage change compared to baseline, instead of absolute values. Given the findings in the 6-month study, analyses were performed for RR and progressive groups separately [21]. For all tests a P < 0.05 was considered significant.

## Results

Sixty PwMS were included in the study and completed the questionnaires at baseline. They received the Can Do treatment in March 2011, April 2011, February 2012, April 2012, June 2012 and August–September 2012; the 1-, 3-, and 6-month data of the March 2011 and April 2011 groups were included in the previous analysis [21]. Three PwMS did not complete the 3-day treatment and were therefore excluded from the analysis. Thirty-eight of the 57 PwMS completed the 12th month questionnaires (response rate 66.7 %), 25 females and 13 males; of these, 22 had RRMS and 14 progressive MS, and in two the disease course was unknown. As a result there were 36 analysable patients. In the first two groups (March 2011, April 2011) the baseline and 12th month assessments were performed via paper questionnaires, whereas in the other groups the baseline data were obtained via paper questionnaires and the 12th month data online.

The mean (SD) values at baseline and at 12 months, with the relative difference (percentage of baseline) and the P value for the comparison, for MSSES Function, MSSES Control, IPA Limitations, IPA Problems, MSQoL-54 Physical, MSQol-54 Mental, HADS Anxiety, HADS Depression and MFIS-5 scores in the RR group are presented in Table 1, and the corresponding values in the progressive group are presented in Table 2.

**Table 1:** Mean (standard deviation) values at baseline and 12 months after the Can Do treatment with relative difference (percentage of baseline) (mean, standard error of the mean) and P value for comparison (paired *t*-test) for self-efficacy [multiple sclerosis self-efficacy scale (MSSES)], impact on participation and autonomy [impact on participation and autonomy questionnaire (IPA)], HRQoL [multiple sclerosis quality of life-54 Items (MSQoL-54)], anxiety and depression [Hospital anxiety and depression scale (HADS)], and fatigue [modified fatigue impact scale-5 items (MFIS-5)] in the relapsing remitting group (N = 22); Δ, difference

Relapsing remitting group	Baseline	12th month	% Δ	P
MSSES function (N = 21)	80.86 (15.00)	79.58 (16.24)	−0.68 (3.00)	0.708
MSSES control (N = 21)	57.68 (19.29)	64.02 (15.81)	+20.21 (7.83)	0.012
IPA limitations (N = 22)	2.40 (0.60)	2.28 (0.60)	−0.53 (7.41)	0.356
IPA problems (N = 20)	1.97 (0.37)	1.75 (0.51)	−8.38 (5.51)	0.075
MSQoL-54 physical (N = 11)	56.87 (11.93)	59.85 (18.68)	+14.99 (7.09)	0.032
MSQoL-54 mental (N = 17)	55.98 (11.82)	63.47 (14.12)	+17.38 (8.16)	0.087
HADS anxiety (N = 22)	7.23 (3.45)	5.50 (3.16)	−25.89 (8.64)	0.044
HADS depression (N = 22)	5.59 (3.62)	3.86 (3.23)	−29.76 (14.53)	0.042
MFIS-5 (N = 22)	12.09 (3.95)	9.95 (3.77)	−19.82 (7.34)	0.080

**Table 2:** Mean (standard deviation) values at baseline and 12 months after the Can Do treatment with relative difference (percentage of baseline) (mean, standard error of the mean) and P value for comparison (paired *t*-test) for self-efficacy [multiple sclerosis self-efficacy scale (MSSES)], impact on participation and autonomy [impact on participation and autonomy questionnaire (IPA)], HRQoL [multiple sclerosis quality of life-54 items (MSQoL-54)], anxiety and depression [Hospital anxiety and depression scale (HADS)], and fatigue [(modified fatigue impact scale-5 items (MFIS-5)] in the progressive group (N = 14); Δ, difference

Progressive group (N = 14)	Baseline	12th month	% Δ	P
MSSES function (N = 13)	56.24 (22.36)	53.65 (25.55)	−5.34 (5.34)	0.438
MSSES control (N = 13)	47.80 (15.00)	52.78 (20.01)	+7.96 (8.15)	0.297
IPA limitations (N = 14)	2.76 (0.68)	2.72 (0.66)	−0.30 (6.45)	0.720
IPA problems (N = 14)	1.90 (0.34)	1.84 (0.46)	−1.58 (6.48)	0.631
MSQoL-54 physical (N = 5)	38.12 (6.46)	45.54 (18.12)	+4.18 (13.05)	0.719
MSQoL-54 mental (N = 10)	53.83 (17.32)	57.03 (14.29)	+8.06 (9.51)	0.649
HADS anxiety (N = 13)	7.21 (3.38)	6.31 (4.55)	−6.99 (12.78)	0.477
HADS depression (N = 14)	6.71 (3.89)	6.50 (3.78)	+25.59 (25.11)	0.843
MFIS-5 (N = 14)	12.57 (4.03)	11.93 (3.36)	−1.00 (6.60)	0.395

Twelve months after the Can Do treatment we observed that in the RR group, compared to baseline, self-efficacy control had increased by 20 % and physical HRQoL by almost 15 %, and that depression and anxiety had decreased by approximately 30 and 26 %, respectively. Moreover, the percentage changes in mental MSQoL-54 and MFIS-5 scores, and the corresponding P values suggested that mental HRQoL (+17 %) and fatigue (−20 %) might also have improved. In contrast, in the progressive group no changes suggestive of improvement were seen, neither with respect to P value, nor regarding the degree of change, except for the 25 % decrease in the depression score (P = 0.843).

## Discussion

In this study we explored whether the Can Do treatment, an intensive social cognitive treatment with support partners, might have long term beneficial effects in PwMS. We previously reported that 6 months after treatment persons with RRMS showed a 25 % increase in self-efficacy control and 22 and 18 % increases in mental and physical HRQoL, respectively. In line with these findings we now observed that in RRMS self-efficacy control and physical HRQoL had increased at 12 months compared to baseline by 20 and 15 %, respectively. With respect to progressive patients, the 6-month study showed no statistically significant changes in self-efficacy or HRQoL, nor in other outcomes. Likewise, in this 12-month study, changes suggestive of both statistical significance and clinical relevance were observed in not a single outcome in the progressive group.

Apart from the P values, the degree at which various scores increased or decreased in the RRMS group, namely by 15–20 % or more, suggests that the 12-month changes may indeed have been clinically relevant and may have been experienced by the PwMS as such: having more confidence with managing symptoms and coping with the demands of their disease, having a better overall well-being, being less anxious, being less fatigued, and having a better mood.

We cannot explain the absence of improvements in progressive patients. However, based on the psychiatrist’s (RR) experience with patients with chronic diseases, like diabetes mellitus, we think that the resilience of progressive MS patients is again and again tried by the continuous increase in disabilities and limitations, and that this may result in a chronic mourning process. In contrast, RRMS patients are not constantly confronted with the consequences of their disease, since they not only experience recoveries from relapses but also stable periods in between relapses. As during the Can Do treatment no differences were observed by the multi-disciplinary team between relapsing–remitting and progressive patients, it might well be that progressive patients need long-term, low-intensity aftercare.

The major limitation of the study is that, given the uncontrolled design, no conclusion can be drawn about a causal relationship between the Can Do intervention and the observed changes. A confounding factor to be considered is a possible change in disease modifying treatment between baseline and the 12th month. For, in line with the underlying concept, the Can Do treatment may well have resulted in a higher assertiveness and a better communication with the neurologist and nurse, leading to the start of (more potent) disease modifying treatment; it has been known that disease modifying treatments may increase HRQoL in RRMS [31]. This confounder cannot have been operative in the progressive group, as no such treatments are available for progressive MS.

Our study has several other limitations. Firstly, the number of patients is rather low, notably in the progressive group. Nevertheless, there is a virtual absence of changes in the progressive group, both with respect to P values and percentage differences (except the depression score), and this contrasts with the findings in the RRMS group (seven out of nine P < 0.10; six out of nine difference ≥15 %), suggesting that indeed eventual beneficial effects of the treatment are limited to RRMS. Secondly, in the last four treatment groups the baseline data were acquired via the paper-and-pencil method, whereas the 12th month data were obtained online. We think that this methodological inconsistency had no major impact on the outcomes, as it has been reported that the quality of web-based data acquisition is equivalent to that of conventional methods [32]. Thirdly, although the 12-month response rate was reasonable, it may well be that those PwMS who were disappointed due to a lack of improvement have preferentially not completed the follow-up questionnaires.

In all, this study suggests that the intensive social cognitive Can Do treatment with participation of support partners might have long lasting beneficial effects on control self-efficacy and HRQoL in persons with RRMS. The findings seem to confirm our choice to perform a randomized controlled trial on the effectiveness of the Can Do treatment specifically in persons with the RR type of disease [33]. This trial is ongoing.

## Abbreviations

CCMO: centrale commissie mensgebonden onderzoek
HADS: hospital anxiety and depression scale
HRQoL: health-related quality of life
IPA: impact on participation and autonomy
METOPP: medisch-ethische toetsing onderzoek patiënten
MFIS-5: multiple sclerosis fatigue impact scale 5-items
MS: multiple sclerosis
MSQoL-54: multiple sclerosis quality of life 54-items
MSSES: multiple sclerosis self-efficacy scale
PwMS: persons with multiple sclerosis
RR: relapsing remitting
RRMS: relapsing remitting multiple sclerosis
P: probability
SD: standard deviation
SEM: standard error of the mean
VPN: virtual private network
WMO: wet medisch-wetenschappelijk onderzoek met mensen
